# A case report of colon interposition radical surgery performed via unilateral thoracotomy in a patient with esophageal cancer after billroth II gastrectomy

**DOI:** 10.3389/fonc.2024.1403192

**Published:** 2024-09-24

**Authors:** Chun-Guang Wang, Guang-Xin Zhang, Yan Zhang, Hang Guo, Guang-Lei Zhang, Zhen-Hua Kang

**Affiliations:** ^1^ Department of Thorax, The Second Hospital of Jilin University, Changchun, China; ^2^ Department of Colorecal & Anal Surgery, The First Hospital of Jilin University, Changchun, China

**Keywords:** BII gastrectomy, colonic interposition, esophageal cancer, thoracotomy, case report

## Abstract

**Introduction:**

When a gastric tube cannot be used as a substitute for the esophagus, the colon offers several advantageous features for esophageal replacement. However, this procedure remains complex and necessitates patients to have a favorable nutritional status. In this study, we investigated the viability of intrathoracic colonic interposition anastomosis through a single thoracic incision, with the goal of mitigating surgical challenges and nutritional requirements.

**Case description:**

We conducted a colectomy and reconstructed the esophageal-colonic-gastric tract via the esophageal bed into the left thoracic cavity for a 68-year-old male patient with compromised nutritional status following 30 years post-Billroth II (BII) gastrectomy. Under normal circumstances, this patient would not have been deemed an appropriate candidate for a conventional colonic interposition procedure. The patient resumed a soft diet through the normal digestive tract two weeks after the surgery and was discharged 20 days later.

**Conclusion:**

Patients who have previously received a Billroth II Gastrectomy and subsequently developed early-stage esophageal cancer, characterized by the absence of lymph node metastasis, are suitable candidates for Colon Interposition Radical Surgery via left thoracotomy.

## Introduction

The use of the stomach as a substitute for the esophagus after esophagectomy in cases of thoracic esophageal cancer is widely accepted as the standard procedure (1). However, for patients with a history of gastrectomy or those with cancer affecting both the esophagus and stomach, reconstruction may require colon segments transposed on a vascular pedicle as a reliable and versatile conduit for esophageal replacement when the stomach is unavailable. Initially described with few reported cases in the first half of the 20th century (2, 3), this procedure gained popularity through the work of Belsey (4), Skinner (5), and Demeester (6). The ascending or descending colon can serve as esophageal substitutes with blood supply from the right colic, middle colic, and left colic arteries. Various techniques have been proposed to increase colonic length or blood circulation for esophageal reconstruction, tailored to different colonic conditions. Compared to gastric tube reconstruction, colon interposition requires a longer operation time for colon mobilization and additional anastomosis, leading to increased surgical stress and postoperative complications (7). A notable drawback is the higher incidence of postoperative leakage and colonic necrosis associated with this method. To address these challenges, we introduce a method involving left transthoracic resection and anastomosis of the esophageal-colonic-gastric conduit, aimed at reducing operative time and minimizing postoperative complications.

## Case presentation

**Figure 1:**
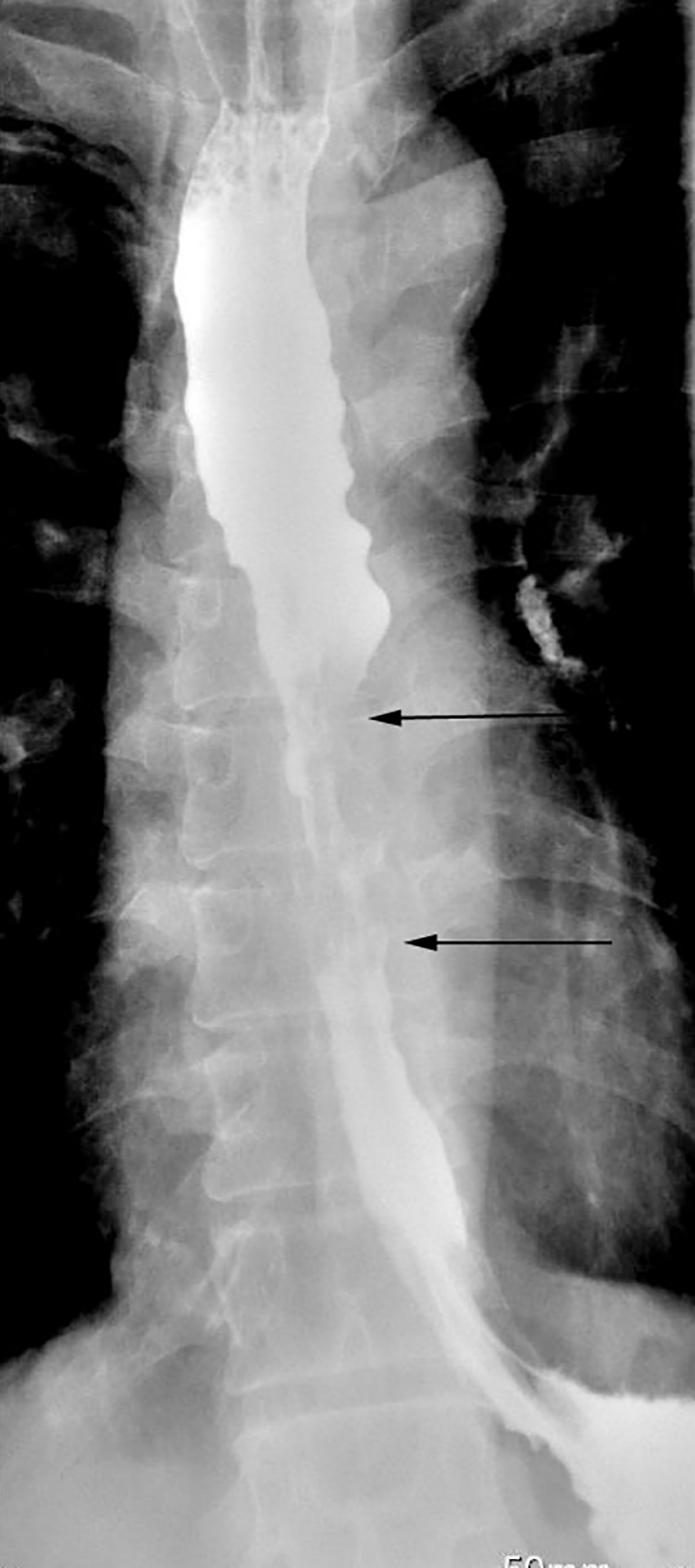
Gastrografin swallow study showing a 5-cm-long irregular, ragged mucosal pattern with annular lunimal narrowing indicated between two arrows with proximal dilation of esophagus.

The patient, a 68-year-old male, had a medical history of distal gastrectomy performed 30 years ago due to gastric perforation. He presented with intrathoracic esophageal cancer and had several risk factors, including smoking one pack of cigarettes daily for over three decades and consuming five ounces of alcohol daily for the same period. He reported progressive dysphagia of solid foods over the past year. Gastrografin fluoroscopy of the esophageal tract revealed significant enlargement of the upper and middle esophagus, with a narrow stream of barium passing through the tumor (**Figure 1**). Esophagogastroscopy indicated a 5.0 cm mass located 30 cm from the dental arch, and biopsy confirmed esophageal squamous cell carcinoma. The PET-CT examination showed there was no metastasis lymph node in thoracic or abdomen cavity and there was no anastomotic leak related to the Billroth II Gastrectomy. Prior to surgery, colonoscopy was performed to rule out the presence of malignant tumors or inflammation in the colon, during which two small masses of adenomatous polyps were cauterized. Angiography was not conducted as the plan was to utilize the transverse and descending colon.

**Figure 2:**
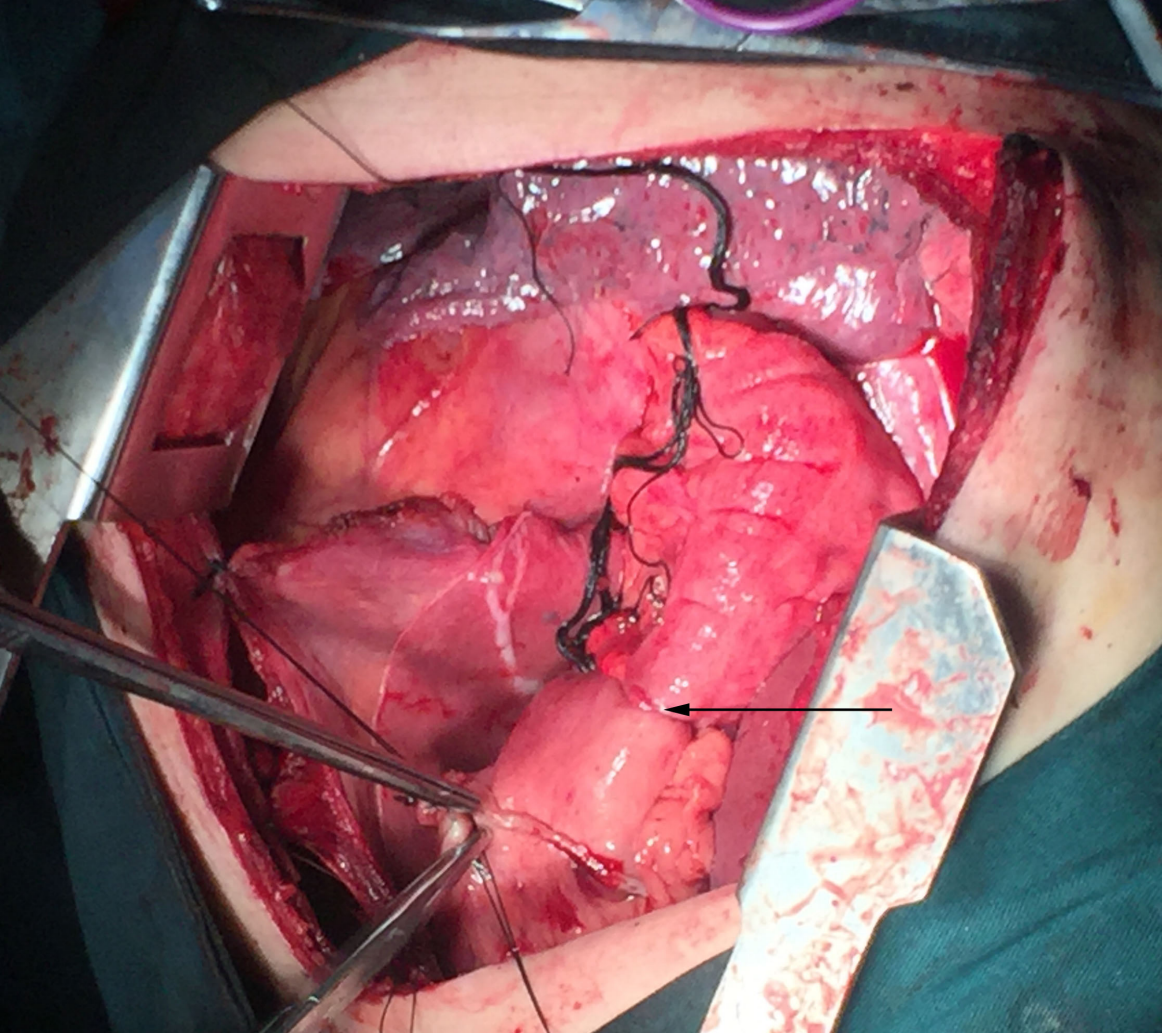
Anastomosis made between the colon and the anterior wall of gastric remnant under the diaphragm in an operative photograph.

A left posterolateral thoracotomy was performed through the sixth intercostal space, and a diaphragmatic incision was made to facilitate colon mobilization and the creation of three anastomoses for colonic interposition. Because of a single thoracic incision, this case is performed a two field nodal dissection. The 107,108 and 110 lymph node was removed in the operation, and the pathological result is negative in lymph node. The previous surgical procedure was Billroth II precolonic gastrointestinal anastomosis. There are no lesions in the colon. The ascending branch of the left colic artery and the left branch of the middle colic artery were well developed. Transverse colon and partial descending colon were selected as the transplanted colon segments. The ascending branch of the left colic artery and the left branch of the middle colic artery were selected as the supply vessels and the mesocolon of the transplanted segment was loosened. Cut off the dissociated area below the cardia with a stapler with a seromuscular suture. The esophagus was separated upward to the subarch and the mediastinal lymph nodes were removed. A purse-string suture was made at the intended cut point of the esophagus, and the anvil of a 25-mm Premium Plus CEEA circular stapler (Covidien Surgical) is inserted. The middle colic artery, transverse colon and its parablimbic arch were isolated. A length of colon was measured, and the descending colon and its parablimbic arch were severed at the distal end of the descending colon at 5cm. The transverse colon was anastomosed laterally to the stumps of the descending colon, and then the stumps of the transverse colon were closed with a seromuscular suture. The proximal end of the transplanted segment was lifted to the thoracic cavity, and the proximal end of the colon was purse-string sutured. The circular stapler is introduced through the choracic incision, carefully placed into the proximal colon conduit, and brought out from the stumps of the transverse colon. The spike and anvil are married and the anastomosis is created, and the esophageal stump was anastomosed with the colon stump below the aortic arch. The distal end of the transplanted colon was sutured with the purse string and placed I nto the anvil. A stoma was made on the posterior wall of the stomach and a stapling device was placed. The distal end of the transplanted colon was anastomosed with the lateral side of the residual anterior wall of the stomach, and then the stoma was closed. (**Figure 2**).

**Figure 3:**
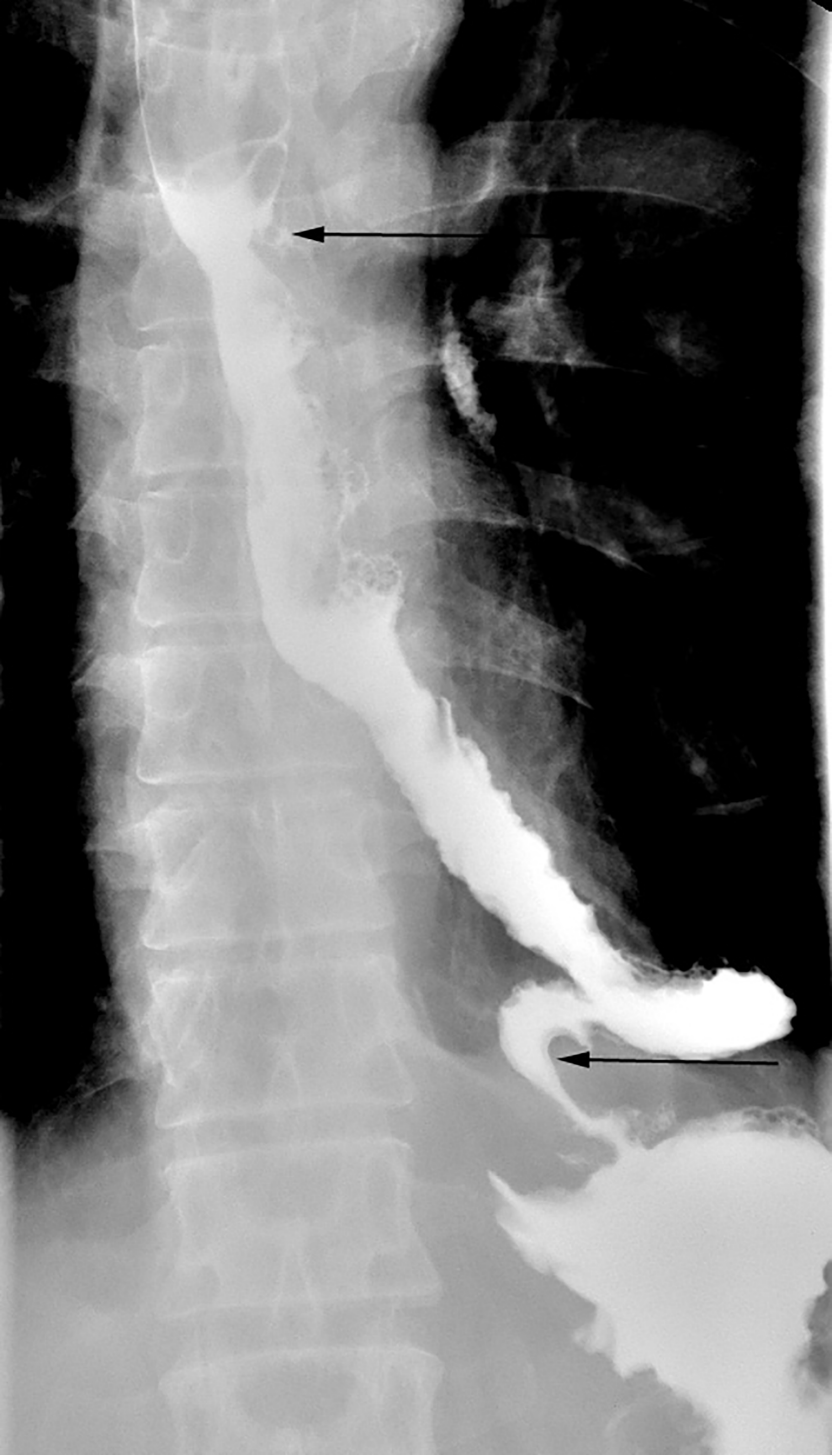
Upper gastrointestinal study after the operation showing the free flow of gastrografin from the upper esophagus to the stomach. The arrows indicate proximal and distal end of interposed colon.

The postoperative pathology report indicated a moderately differentiated esophageal squamous cell carcinoma. The tumor, measuring 5.0 x 2.5 cm, was classified as a medullary-type lesion that had penetrated beyond the muscularis externa. Lymph node examination did not reveal any metastasis. The esophageal cancer was staged as a moderately differentiated mid-thoracic squamous cell carcinoma with a tumor node metastasis (TNM) stage of pT3N0M0. Two weeks following the surgery, the patient was allowed to consume fluids. There were no complications directly related to surgery, such as chylothorax, thoracic empyema, and cardiac arrhythmias. One month post-surgery, a follow-up gastrografin swallow study demonstrated no elongated or tortuous colon in the left thorax (**Figure 3**), with the contrast material freely passing into the stomach through the reconstructed colon.

## Discussion

The stomach is commonly preferred as the primary choice for esophageal replacement following esophagectomy, while colonic interposition is frequently utilized as an alternative when the stomach is not an option. Various approaches to transthoracic esophageal resection involving the stomach include Ivor Lewis with high intrathoracic anastomosis (requiring laparotomy and right thoracotomy), McKeown with cervical anastomosis (involving right thoracotomy, laparotomy, and neck incision), and Sweet with high intrathoracic anastomosis (performed as left transthoracic esophagectomy).Standard radical surgical resection for thoracic esophageal carcinoma typically involves either Ivor Lewis esophagectomy or McKeown esophagectomy, both of which entail a two-field lymphadenectomy. However, randomized trials have failed to demonstrate a significant difference in 5-year survival rates between these approaches, although McKeown esophagectomy has been associated with increased postoperative morbidity compared to Ivor Lewis (8, 9). While Ivor Lewis esophagectomy and McKeown esophagectomy have been considered superior surgical resections due to their extensive lymphadenectomy compared to Sweet esophagectomy, there is ongoing debate regarding the prognostic benefits of these approaches and which surgical method achieves the best balance between radical resection, postoperative outcomes, and survival. The left transthoracic approach is not inferior to the Ivor Lewis procedure in terms of efficiency regarding lymphadenectomy, postoperative complications, and long-term survival. Additionally, the Sweet procedure is deemed safer due to its simpler operation process, minimal patient trauma, and fewer complications after surgery, except for the extensive removal of lymph nodes. In some respects, the Sweet procedure is even considered superior to the right transthoracic approach regarding surgical and oncological outcomes in the treatment of patients with negative lymph nodes (10, 11). Colonic interposition is a sophisticated surgical technique that should be considered among the skills of esophageal surgeons. Despite being performed in experienced medical facilities, it can result in considerable morbidity and mortality, particularly when compared to procedures like the Ivor Lewis esophagectomy or the McKeown esophagectomy. A recent meta-analysis involving more than 1,849 patients, conducted by Brown (12) revealed a mortality rate of 7.8%, morbidity rate of 13.6%, and a leak rate of 11% associated with colonic interposition, underscoring the necessity for meticulous planning and a multidisciplinary approach. Although esophago-colonic anastomosis is typically performed in the neck, it can also be conducted in the chest. Recently, Gooszen (13) reported a lower incidence of anastomotic leakage (17% versus 21.9%) in patients with intrathoracic anastomosis compared to those with cervical esophagogastrostomy. This suggests that the transthoracic approach with an intrathoracic anastomosis may be safer and more beneficial, particularly for patients with carcinoma of the lower and middle third of the esophagus, due to reduced rates of anastomotic leakage, wound infection, recurrent laryngeal nerve paresis, and shorter hospital stays. However, compared to gastric pull-up, colonic interposition is associated with longer operating times due to colon mobilization and additional anastomosis, increasing the risk of complications (14). Overall, morbidity rates appear to be significantly higher with colonic interposition, necessitating careful consideration of reconstruction methods, with the retrosternal route being a common choice to mitigate the risk of severe infection and sepsis resulting from anastomotic leakage or colon graft necrosis within the posterior mediastinum (14–17).

Given that the Sweet transthoracic approach demonstrates comparable long-term survival to the Ivor Lewis procedure and offers enhanced safety with its simpler operation process, minimal patient trauma, and reduced postoperative complications, it is justified to contemplate the adoption of left thoracotomy and intrathoracic anastomosis. This consideration is aimed at circumventing complex and prolonged surgical interventions and their potentially severe complications. The primary criteria for selecting either a pulled stomach or transplanted colon as an esophageal substitute are ensuring an adequate blood supply and sufficient length, which can be particularly challenging, especially for neck anastomosis. Preoperative angiography or examination of the vascular blood supply through palpation and transillumination of the mesentery (18) can help determine the preferred esophageal substitute—either the left colon or the right colon. Peters (19) conducted a study involving 25 patients who underwent colonic interposition, each of whom underwent mesenteric angiography prior to the procedure. The results indicated that 12% of the patients had multiple middle colic arteries, while 96% had an ascending branch of the left colic artery. Consequently, the left colon was chosen due to its demonstrated reliability in terms of blood supply as supported by anatomical studies. Additionally, its mobilization was feasible through the left thoracic incision, which was suitable in our case. In cases where the operation did not progress as expected, the option of supercharging colonic conduits was considered as an alternative approach. This technique was first introduced by Longmire in 1946, but it gained widespread acceptance only after O’Rourke published a series of 14 cases in 1985, demonstrating successful outcomes without anastomotic leaks or conduit ischemia. While most published studies report a low incidence of conduit ischemia with supercharging, the limited number of patients included in these studies prevents definitive conclusions from being drawn.

Considering adequate length, opting for left thoracotomy and intrathoracic anastomosis reduces the required length of the colon and its associated artery and vein, thus decreasing surgical complexity and lowering the risk of anastomotic tension and subsequent leakage. This approach also eliminates the need for laparotomy and cervicotomy, as colonic mobilization and anastomosis are attainable via the diaphragmatic incision. This is particularly advantageous for patients presenting with poor nutritional status, thereby enhancing postoperative recuperation. In addition to conventional colic interposition, some scholars advocate for ileocolic interposition, where the ileocecal artery is transected proximally to create a long segment of terminal ileum, ranging from 15 to 20 cm, suitable for reaching the neck (20). In a retrospective study of 30 patients utilizing isoperistaltic right colon pedicled from the middle colic artery, Oida et al. (21) compared posterior mediastinal and subcutaneous conduits and found that more cases in the subcutaneous group required supercharging due to ischemia and had higher rates of anastomotic leaks. They propose that the shortest route may improve perfusion, reduce tension, and lower the risk of ischemia and leak. Other scholars advocate for supercharged (22) or super drainage (23) ileocoloplasty, a microsurgical technique that enhances vascularization by anastomosing the ileocolic artery or vein to a vessel in the neck, especially in patients with a short esophageal remnant or high ischemia risk. Despite the advantages of various colonic interposition methods, they entail complex surgical procedures, including microvascular techniques, posing a risk of morbidity and mortality. In discussing the differences between the Ivor Lewis approach, Sweet approach, and Modified Sweet approach regarding upper mediastinal lymph nodal dissection, firstly, Patient-specific factors, such as comorbidities, physical constitution, and personal preferences, should be taken into account. Second, The Ivor Lewis esophagectomy involves an abdominal incision and a right thoracotomy for access to the esophageal tumor, with the anastomosis performed in the upper chest. With the advent of the robotic era in intrathoracic Ivor Lewis Esophagectomy (24), robotic-Sewn Anastomosis may become minimally invasive and feasible due to its clear surgical field of view and its multi-axis operating system. In contrast, the Sweet approach and its modifications may offer different angles of access and visualization, which could impact the lymph nodal dissection. Each approach may provide varying degrees of access to specific lymph node stations in the upper mediastinum. The extent of dissection and the number of lymph nodes that can be safely and effectively removed may differ between these techniques.

However, left thoracotomy also comes with its drawbacks. First, Ivor Lewis surgery can remove more lymph nodes than Sweet surgery, and patients maybe have better long-term survival benefits without increasing postoperative complications; Compared with Ivor Lewis, Sweet surgery is more convenient, less time-consuming and well tolerated by patients. Second, For instance, there is a lack of reported cases in current literature regarding colonic interposition via separate left thoracotomy, leaving surgeons with no prior experience to draw upon when planning the surgery for the patient. Third, another concern is the potential risk of fatal infection if an anastomotic fistula were to occur, given that colonic secretions contain Escherichia coli, unlike the germ-free conditions found in the stomach. Additionally, one disadvantage of using a single incision is its limited applicability, primarily suited for lower esophageal and cardiac cancer cases without clear lymph nodal metastasis due to restricted lymph nodal resection in the thorax and abdomen.

While performing esophagectomy and colon interposition via a single thoracic may lessen surgical complexity, it remains a technically demanding procedure in adult patients. It requires the expertise of the thoracic surgeon, often involving collaboration with colorectal surgeons, and occasionally with plastic microvascular surgeons for cases requiring supercharged conduits.

## Conclusion

Our clinical practice and case reports demonstrate that for patients who have undergone Billroth II Gastrectomy and concurrently developed early esophageal cancer without lymph node metastasis, it is feasible to perform Colon Interposition Radical Surgery through a unilateral thoracic approach, thereby achieving curative treatment for this specific group of patients. This approach has been validated by our experience and the outcomes we have reported, showcasing its effectiveness in managing early esophageal cancer in such unique clinical scenarios.

## Data Availability

The original contributions presented in the study are included in the article/supplementary material, further inquiries can be directed to the corresponding author/s.
